# Erythropoietin-producing hepatocellular receptor B6 is highly expressed in non-functioning pituitary neuroendocrine tumors and its expression correlates with tumor size

**DOI:** 10.1007/s11033-023-09186-7

**Published:** 2024-02-11

**Authors:** Hadara Rubinfeld, Zvi R. Cohen, Uzi Bendavid, Suzana Fichman-Horn, Adva Levy-Barda, Cfir David, Philippa Melamed, Ilan Shimon

**Affiliations:** 1 Institute of Endocrinology, Diabetes & Metabolism and Felsenstein Medical Research Center, Rabin Medical Center, Beilinson Campus, 49100 Petach Tikva, Israel; 2 Department of Neurosurgery, Sheba Medical Center, Tel-Hashomer, Israel; 3 School of Medicine, Tel Aviv University, Tel Aviv, Israel; 4 Department of Neurosurgery, Rabin Medical Center, Petah Tikva, Israel; 5 Department of Pathology, Rabin Medical Center, Petah Tikva, Israel; 6 Biobank, Department of Pathology, Rabin Medical Center, Petah Tikva, Israel; 7 Faculty of Biology, Technion – Israel Institute of Technology, Haifa, Israel; 8 Faculty of Medicine, Tel Aviv University, Tel Aviv, Israel

**Keywords:** EphB6, Ephrins, Pituitary, NF-PitNETs, RTK

## Abstract

**Background:**

Erythropoietin-producing hepatocellular (EPH) receptors are the largest known family of receptor tyrosine kinases characterized in humans. These proteins are involved in tissue organization, synaptic plasticity, vascular development and the progression of various diseases including cancer. The Erythropoietin-producing hepatocellular receptor tyrosine kinase member EphB6 is a pseudokinase which has not attracted an equivalent amount of interest as its enzymatically-active counterparts. The aim of this study was to assess the expression of EphB6 in pituitary tumors.

**Methods and Results:**

Human normal pituitaries and pituitary tumors were examined for EphB6 mRNA expression using real-time PCR and for EphB6 protein by immunohistochemistry and Western blotting. EphB6 was highly expressed in non-functioning pituitary neuroendocrine tumors (NF-PitNETs) versus the normal pituitary and GH-secreting PitNETs. EphB6 mRNA expression was correlated with tumor size.

**Conclusions:**

Our results suggest EphB6 aberrant expression in NF-PitNETs. Future studies are warranted to determine the role and significance of EphB6 in NF-PitNETs tumorigenesis.

## Introduction

Ephs are the largest family of receptor tyrosine kinases (RTK). Ephs orchestrate cell positioning as well as tissue and organ patterning. Ephs also control cell survival during normal and neoplastic development and they have been implicated in cancer cell proliferation, adhesion, migration, tumor angiogenesis and invasion [1–3]. A unique feature of the Eph receptors is that their cognate ligands, the ephrins, are tethered to the cell surface, in contrast to other RTKs whose ligands are generally soluble [4]. Therefore, the resultant signaling is largely dependent on cell–cell contact and can occur in a bidirectional manner in neighboring cells [4]. Eph receptors are divided into two subfamilies, types A and B. Whereas EphA and EphB receptors have a similar structure, the structures of the ligand classes, ephrin-A and -B, are different. Ephrin-B are transmembrane ligands while ephrin-A ligands are smaller and tethered to the membrane only via a glycosylphosphatidylinositol (GPI) anchor [5].

Two members of the family, EphA10 and EphB6, are classified as pseudokinases due to the absence of key amino acids known to catalyze phosphoryl transfer from ATP in conventional kinases [6, 7]. Yet, these two receptors are able to function without tyrosine kinase activity. EphB6 was shown to maintain physiological homeostasis in kidney [8], vascular smooth muscle [9] and the immune system [10]. However, considerably more research was focused on EphB6 involvement in cancer. EphB6 was shown to reduce motility and invasion of breast [11–13] and lung [14, 15] cancer cells. In several malignancies, an inverse correlation between EphB6 expression and tumor aggressiveness was observed thereby suggesting that EphB6 may suppress invasive and metastatic phenotypes [16–26]. Interestingly though, despite its anti-invasive properties, EphB6 was found to promote tumor initiation in breast cancer xenografts [12] and in a colorectal cancer model [27, 28]. Consistent with this oncogenic potential of EphB6, its expression was positively correlated with tumor size and recurrence rate of malignant thyroid lesions [29] and was also coupled to poor outcome in breast cancer [30] and tongue squamous cell carcinoma [31].

Interestingly, Eph receptor/ephrin signaling is known to play important roles in various niches and was investigated also in the context of the normal physiology of the pituitary gland [32–34]. Prolactin secreting pituitary neuroendocrine tumors (PitNETs) are the most common pituitary tumors (50%) followed by non-functioning pituitary neuroendocrine tumors (NF-PitNETs) comprising ~ 30% of PitNETs [35]. Dysregulated expression of Eph family members in NF-PitNETs was reported or may be obtained from several reports as part of broad gene expression profiling of PitNETs [36–45]. In this study we aimed to examine the expression of EphB6 in PitNETs.

## Materials and methods

### Pituitary tumors

Samples of human pituitary tumors were obtained during transsphenoidal surgical resection with patients' informed consent in accordance with methods and conditions approved by the local institutional review board (approval number 0838-17). 30 NF-PitNETs and 17 GH-PitNETs were analyzed in this study. The clinical characteristics of the patients are presented in Table 1.

**Table 1:** Clinical characteristics of patients with PitNETs

Tissue type	Size (mm)	Age (yr)/gender	Histologic diagnosis	Experiment
*NF-PitNETs*				
NF1	9	31/F	FSH	qPCR
NF2	14	54/M	FSH, scattered β-LH	qPCR
NF3	15	69/M	Scattered PRL and TSH	qPCR
NF4	16	66/F	FSH, β-LH	qPCR
NF5	16	44/F	FSH, β-LH	qPCR
NF6	18	19/F	NA	qPCR
NF7	18	68/M	FSH, scattered β-LH	qPCR
NF8	20	61/M	FSH, focal TSH	qPCR
NF9	20	74/M	FSH	qPCR
NF10	20	49/M	FSH	qPCR
NF11	25	58/M	FSH, β-LH + focal TSH	qPCR
NF12	28	62/F	FSH, β-LH	qPCR
NF13	29	67/M	FSH, β-LH	qPCR
NF14	32	47/M	FSH, β-LH	qPCR
NF15	37	66/M	foci of FSH, β-LH	qPCR
NF16	44	40/M	Rare cells express FSH	qPCR
NF17	macro	66/M	Focal FSH	IHC
NF18	9	41/F	FSH	IHC
NF19	micro	44/F	FSH	IHC
NF20	macro	43/M	Negative	Western
NF21	40	39/M	FSH, β-LH	Western
NF22	macro	67/F	FSH	Western
NF23	macro	70/F	FSH	Western
NF24	macro	61/F	Focal FSH	Western
NF25	macro	58/M	Negative	Western
NF26	macro	56/M	Negative	Western
NF27	macro	72/M	Negative	Western
NF28	macro	67/M	Focal FSH	Western
NF29	macro	65/M	Focal FSH, β-LH in few cells	Western
NF30	17	64/F	FSH, focal β-LH	qPCR + Western
*GH-PitNETs*				
GH1	macro	66/M	GH	qPCR
GH2	7	51/M	GH	qPCR
GH3	14	37/M	GH, PRL	qPCR
GH4	17	25/F	GH, scattered ACTH	qPCR
GH5	macro	54/F	GH, PRL	qPCR
GH6	13	46/M	GH, scattered TSH, focal PRL	qPCR
GH7	24	54/M	GH, scattered PRL	qPCR
GH8	13	25/M	GH, scattered PRL	qPCR
GH9	15	35/M	GH, PRL	IHC
GH10	macro	57/F	GH, scattered PRL, TSH and β-LH	IHC
GH11	micro	62/F	GH, PRL and TSH in some cells	IHC
GH12	micro	44/M	GH, scattered PRL	IHC
GH13	17	45/M	GH, PRL and TSH in few cells	Western
GH14	22	22/M	NA	Western
GH15	micro	52/M	GH, PRL	Western
GH16	macro	43/M	GH, scattered TSH and FSH	Western
GH17	micro	54/M	GH, PRL in few cells	Western

*NF-PitNETs* non-functioning pituitary neuroendocrine tumors, *GH-PitNETs* growth hormone pituitary neuroendocrine tumors, *NA* not available, *M* male; *F* female, *micro*  < 10 mm, *macro * > 10 mm, *IHC* Immunohistochemistry, *qPCR* quantitative PCR

### Mice

Pituitaries were extracted from GRIC-GFP or GRIC-tdTomato mice (a kind gift from Prof. Ulrich Boehm to Prof. Philippa Melamed) and gonadotropes were enriched by FACS (as in refs.[46, 47]). Animal experiments were performed after protocol approval by the Institutional Animal Care and Use Committee of Technion – Israel Institute of Technology (approval number IL0440415).

### Gene expression

Total RNA was extracted from pituitary specimens and processed to cDNA with High Capacity cDNA Reverse Transcription kit (AB Applied Biosystems, Foster City, CA). Human normal adult pituitary RNA samples of two donors were purchased from BioChain. Human pituitary samples were analyzed in triplicates using Taqman gene expression assays (IDT, Coralville, Iowa, United States). Results were normalized to Cyclophilin B. qPCR reactions of mice samples were performed with PerfeCTa SYBR Green FastMix (Quanta) and normalized to Rplp0. EphB6 mouse primers were: Forward 5` CTAGGAAAGATCTGCGAGGTG 3`, Reverse 5` GTTTGCTCTCTTCATTTACTCTGC 3`.

### Immunohistochemistry

Formalin-fixed, paraffin-embedded 5μ sections slides of pituitary tumors and normal pituitary (obtained from autopsy) were deparaffinized, rehydrated and boiled in citrate buffer. After washing and blocking slides were incubated at 4C with anti-EphB6 antibody (Bioss Antibodies Inc. (Woburn, MA, USA)) diluted 1:200 in PBS. Slides were washed and incubated with a secondary antibody Goat anti-rabbit Alexa Fluor 647 diluted 1:1000 in PBS. After additional washes, slides were stained with Dapi, washed and mounted. Images were obtained with Zeiss ApoTome.2 microscope and scored by ImageJ Fiji software. Setting was consistent for all samples in both image capture and analysis.

### Protein extraction and western blotting

Frozen tissue specimens were minced in liquid nitrogen followed by homogenization in RIPA buffer together with protease and phosphatase inhibitors cocktails. Protein concentrations were quantified with Bradford protein assay (Bio-Rad Protein Assay Dye Reagent Concentrate, Bio-Rad Laboratories, Hercules, CA, USA). Equal amounts of protein extracts were loaded on 10% SDS-PAGE and Western blotting was conducted. Immunodetection was performed using Chemiluminescent Peroxidase Substrate. The optical density of the bands was measured and quantified employing the iBright Imaging System (ThermoFisher Scientific). Antibody against EphB6 was from Santa Cruz Biotechnology Inc. (Dallas, TX, USA).

### Statistical analysis

For independent data Student’s t-test was performed. Correlations were calculated using Pearson test with the GraphPad software. *P* values < 0.05 were considered significant.

## Results

### Elevated EphB6 mRNA expression in NF-PitNETs

**Fig. 1:**
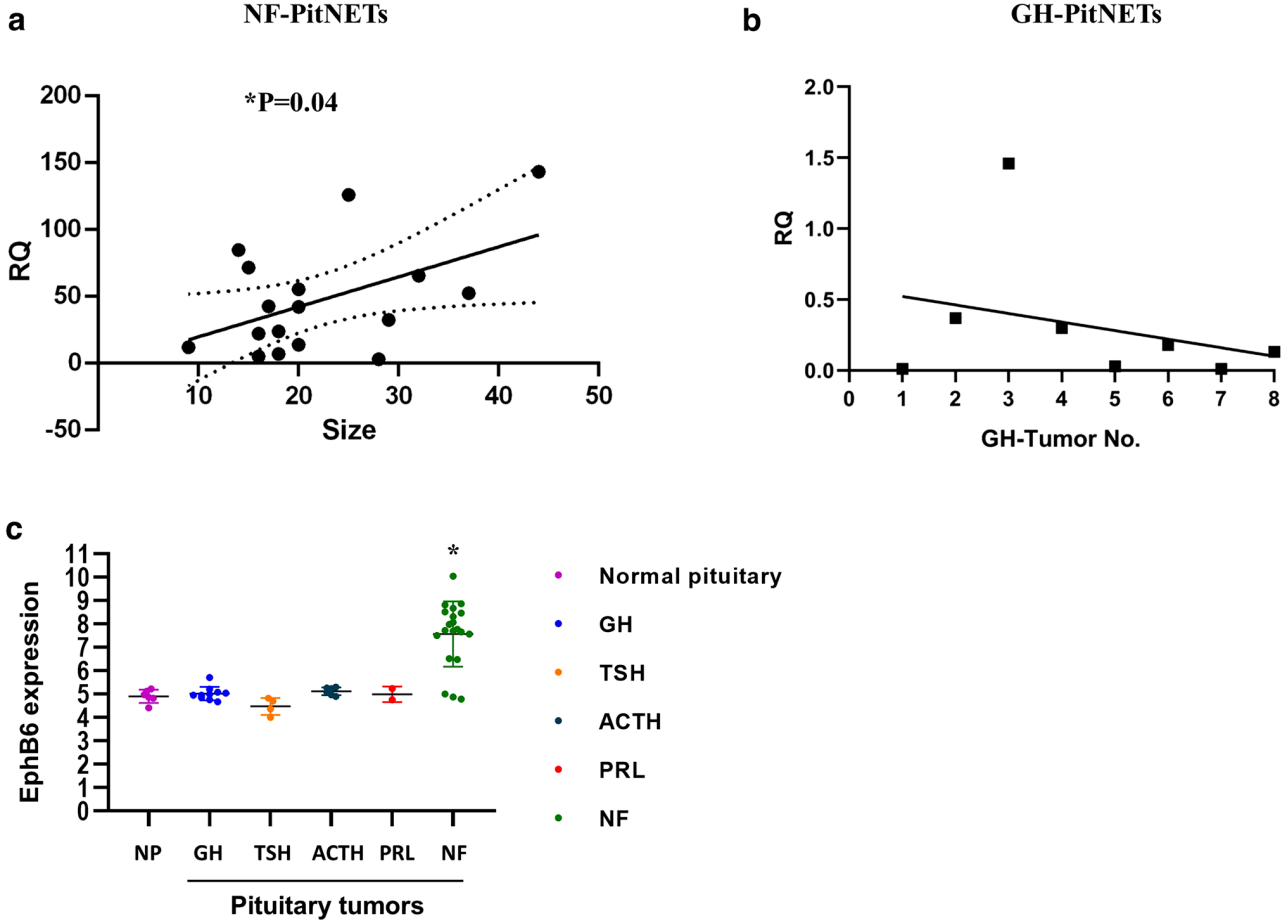
EphB6 mRNA expression. **a** EphB6 gene expressions in NF-PitNETs samples were measured by quantitative RT-PCR and are shown versus normal pituitary. *P = 0.04 for correlation of EphB6 expression level and tumor size. **b** EphB6 gene expressions in GH-PitNETs samples were measured by quantitative RT-PCR and are shown versus normal pituitary.** c** PitNETs were analyzed by Microarray GeneChips [43] and data were obtained from GEO accession GSE147786. Statistical analysis was performed by ANOVA with Tukey post-hoc. *P < 0.0001 for NF-PitNETs vs. normal pituitary, GH, TSH and ACTH PitNETs. *P < 0.0099 for NF-PitNETs vs. PRL PitNETs

We first aimed to validate the high expression of EphB6 mRNA in NF-PitNETs that was found by Moreno CS et al. using gene arrays [39]. Quantitative PCR was performed and revealed that EphB6 mRNA level was significantly higher in all 17 NF-PitNETs samples compared to human normal pituitary and notably was correlated with the tumor size (Fig. 1a). We also examined GH-secreting PitNETs, which showed lower levels of EphB6 mRNA in 7 of 8 samples compared to the normal pituitary (Fig. 1b). Astonishingly, data obtained from Gene Expression Omnibus (GEO;[48], accession GSE147786) of PitNETs analyzed by Microarray GeneChips by Taniguchi‑Ponciano et al.[43], showed that all other types of PitNETs express low levels of EphB6 (Fig. 1c). Finally, since most NF-PitNETs are originated from gonadotropes cells, we aimed to examine if EphB6 dysregulated expression is a marker of normal gonadotropes or specific to tumors. For this purpose, we used genetically modified mice which express GFP in their gonadotropes (gonadotropin-releasing hormone receptor -IRES-Cre (GRIC)-GFP mice [49]). Gonadotropes were collected based on their GFP fluorescence by fluorescence-activated cell sorting at the neonatal period, after castration or when matured and primary cultures were prepared. Quantitative PCR was then performed using αT3 gonadotrope cell line as positive EphB6 control. EphB6 was not expressed in all these primary gonadotropes populations (Ct = O, data not shown). Together, these results suggest that EphB6 aberrant expression is unique to NF-PitNETs.

### Elevated EphB6 protein expression in NF-PitNETs

**Fig. 2:**
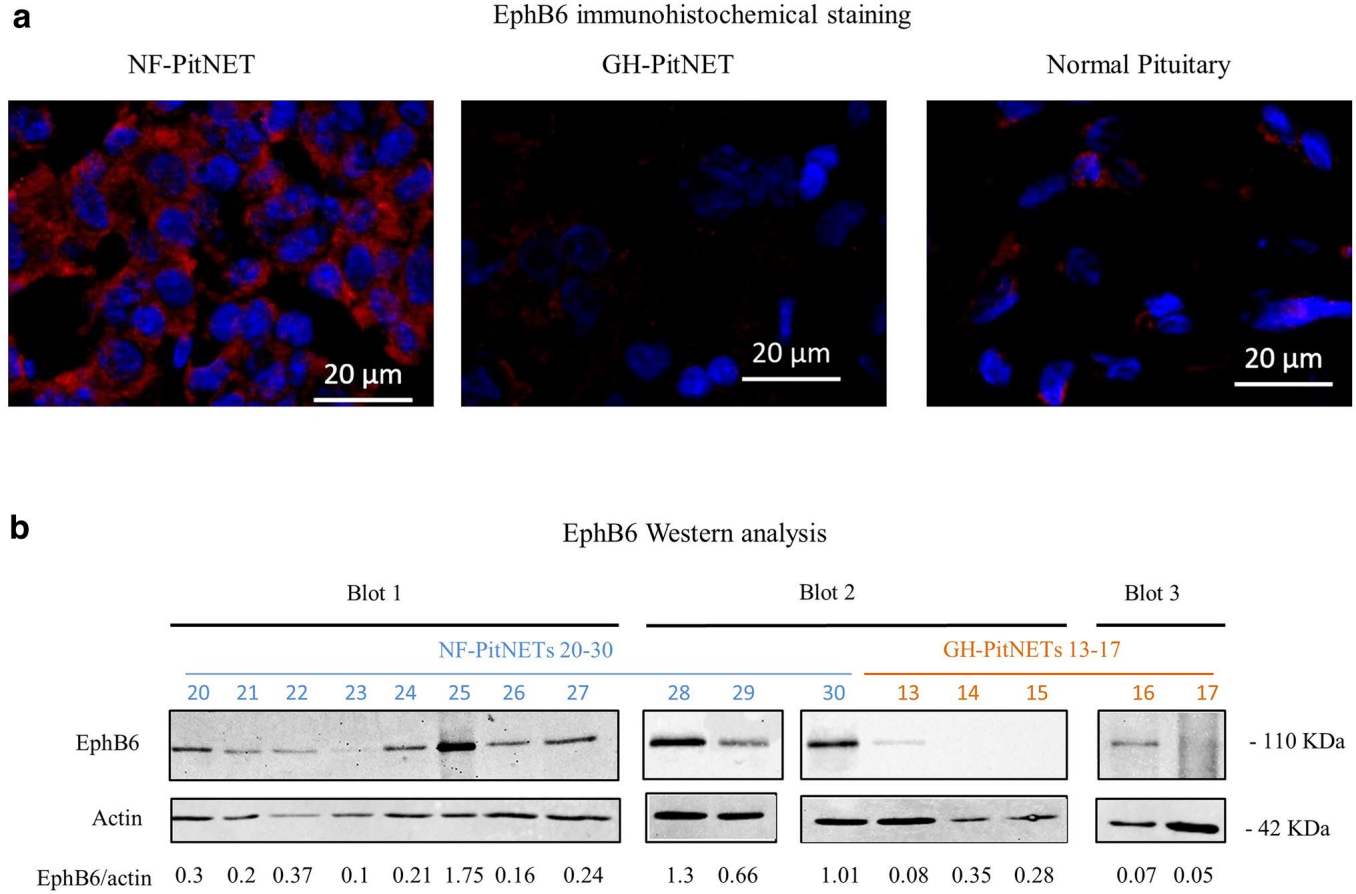
EphB6 protein expression. **a** Immunohistochemical staining of EphB6 in pituitary specimens. Blue-Dapi. Red-EphB6. Images were obtained with Zeiss ApoTome.2 microscope and quantified with ImageJ Fiji software **b** EphB6 Western-blot analysis in NF-PitNETs and GH-PitNETs samples. Samples which did not show actin expression were excluded

We next sought to determine EphB6 protein expression. This was examined by immunohistochemistry of human tissue sections derived from normal pituitary and PitNETs specimens. While the normal pituitary and GH-secreting PitNETs showed low staining, NF-PitNETs exhibited high EphB6 expression (Table 2). Representative immunostained sections of NF-PitNET, GH-secreting PitNET and normal pituitary are shown in Fig. 2a. We also performed Western blot analysis of lysates derived from human PitNETs samples. NF-PitNETs showed significant higher expression levels of EphB6 protein expression compared to GH-secreting PitNETs (Fig. 2b, P = 0.043).

**Table 2:** EphB6 protein expression in human NF-PitNEts and GH-PitNEts

Tissue	% of EphB6 immunopositive cells^a^	Intensity % Area^b^
NF17	59	36
NF18	62	33
NF19	81	36
GH9	1	0.9
GH10	7	5
GH11	20	1
GH12	39	12
Normal Pituitary	21	3

^a^*p* < 0.01

^b^*p* < 0.001 for comparison of NF-PitNEts with GH-PitNEts

## Discussion

Studies investigating the development of the rat pituitary gland have shown co-localization of ephrin B2 ligand (EFNB2) and EphB3 receptor in stem/progenitor cells in the two niches of the anterior lobe, the marginal cell layer and dense cell clusters in the parenchyma [32, 33]. Other EFNB2-candidate interacting receptors, EphB1, EphB2, and EphB4 were found specifically in the rat gonadotropes, corticotropes and endothelial cells, respectively [33]. Also, various ephrin ligands were found to be expressed in the rat anterior pituitary cells by single-cell RNA sequencing [50]. As for EphB6, data obtained from single-cell RNA sequencing studies [51, 52] suggest low EphB6 expression in human fetus gonadotropes which becomes undetectable in adult gonadotropes. In consistent with this, our results show undetectable EphB6 expression in mice gonadotropes populations at the neonatal period and when matured. Together it seems that EPH family members may play a role in the pituitary development and EphB6 is not a marker of the normal gonadotropes subtype.

Profiling studies of NF-PitNETs suggest molecular alterations in several EPH family members. EphB6 [39] and Ephrin-B3 ligand (EFNB3) [36, 39, 41] were found to be overexpressed in NF-PitNETs compared to normal pituitary in microarrays [39, 41] or the GEO database [36]. Moreover, data obtained from RNA sequencing analysis showed a 5.3 fold elevated expression of EphB6 in 43 NF-PitNETs samples compared to 22 functioning PitNETs [42] (GEO;[48], accession GSE209903). In-silico analysis of twenty-three microarray libraries also revealed high expression of EphB6, EFNB3 and other Ephrin receptors, EphA5, -A7, -A10 and -B1 in NF-PitNETs [44]. Quantitative proteomics using two dimensional liquid chromatography-tandem mass spectrometry revealed the expression of EphA10, EFNA5 and EFNB1 in NF-PitNETs [36]. Comparison of highly proliferative NF-PitNETs versus NF-PitNETs suggested differential gene expression of EPH receptor signaling pathway [45] which similarly was found hypomethylated in re-intervention versus stable NF-PitNET patients [37]. Transcriptome analysis of PitNETs identified upregulation of EphB6 in silent ACTH PitNETs, gonadotrophinomas and null cell PitNETs when clustered together and of EphA4 in ACTH-secreting PitNETs [43]. Taken together, these studies and our indicate that NF-PitNETs are characterized by aberrant expression of EphB6 and other EPH receptors and ligands.

In a manner similar to other pseudokinases, EphB6 was suggested to act as a molecular switch that is capable of modulating the signals generated by an Eph receptor cluster. By recruiting kinases, phosphatases, proteases or ubiquitinase ligases (directly or indirectly) EphB6 can modulate the phosphorylation state and thus kinase activity of individual members in the cluster [53]. For example, EphB6 interaction with EphA2 [54–56] and EphB2 [55] was shown to modulated their activities. EphB6 was shown to be phosphorylated by the EphB4 receptor and this tyrosine phosphorylation was crucial for EphB6 interaction with the ubiquitinase ligase c-Cbl and phosphorylation of c-Cbl partner, the Abl kinase [13]. EphB6 can be phosphorylated also by EphB1 [57] or by the Src family kinase Fyn [58]. EphB6 ligands are ephrin-B1 [57] and ephrin-B2 [59] and MAPK [12, 15, 60, 61] and Akt signaling pathways [27, 54, 62, 63] were shown to mediate EphB6 activities. A most recent study by Hanover et al. [64] which integrates bioinformatic analysis, proteomics and genomics reveals crosstalk of EphB6 and EGFR, enhancing the proliferation of cancer cells. Both PI3K/Akt/mTOR and Raf/MEK/ERK signaling pathways downstream to EGFR are activated in NF-PitNETs (reviewed in [65]), therefore a possible EphB6 and EGFR crosstalk in NF-PitNETs is appealing and yet to be investigated.

## Conclusion

Our study show high expression of EphB6 mRNA and protein in NF-PitNETs compared to normal pituitary and GH-secreting PitNET. EphB6 mRNA level was correlated with tumor size. These findings suggest EphB6 as an attractive candidate for functional and clinical studies of NF-PitNETs.

## Data Availability

Not applicable.

## Abbreviations

NF-PitNETs: Non-functioning pituitary neuroendocrine tumors
GH-PitNETs: Growth hormone secreting pituitary neuroendocrine tumors
EPH: Erythropoietin-producing hepatocellular
EGF: Epidermal growth factor
